# “The impact of COVID-19 on employee productivity and the future of remote work”

**DOI:** 10.1371/journal.pone.0351968

**Published:** 2026-06-17

**Authors:** Sultan Alenazi, Jagannath Mohanty, Maheswaran Srinivasan, Durga Prasad Samontaray

**Affiliations:** 1 College of Business Administration, King Saud University, Riyadh, Saudi Arabia; 2 Soil School of Business Design, Manesar, Haryana, India; 3 Institute of Management Technology, Nagpur, Maharashtra, India; 4 College of Business Administration, King Saud University, Riyadh, Saudi Arabia; Universidade Europeia, Lisboa, PORTUGAL

## Abstract

**Purpose:**

This paper explores the impact of the COVID-19 pandemic on employee productivity and the future of remote work beyond the pandemic years. Using data collected from 646 remote workers, we examined the influence of interpersonal relations, time management, stress, and skill development on productivity.

**Design/methodology/approach:**

We validated our model using structural equation modelling (SEM), and the results revealed that the sudden shift to remote work had no significant negative impact on productivity.

**Findings:**

The results of this study may encourage HR managers to expand remote work opportunities, thereby enhancing talent attraction and retention in a highly competitive job market.

**Originality/value:**

Despite prevalent concerns about COVID-19 and the evolving nature of workplaces, employees perceived remote work as offering greater flexibility in task organization and the opportunity to create a healthier work-life balance. Furthermore, our findings suggest that HR managers should consider allowing remote work post-pandemic as a strategic aspect of workplace evolution.

## 1. Introduction

Some businesses have expanded their remote work scope beyond the COVID-19 pandemic [1]. With many organizations having implemented virtual work options since the pandemic, the debate over employee productivity is likely to persist beyond the pandemic. Numerous studies have examined the effects of working remotely [2]. The Gallup survey results published in June 2022 showed that over 60% of employees prefer remote work [3]. Similarly, another survey conducted among 10,646 knowledge workers found that a hybrid model was preferred globally [4]. However, since the onset of the pandemic, most studies have focused their research on job characteristics and employee well-being. Few studies have explored employee productivity in the new working environment.

Many researchers have proposed that remote work enhances productivity by providing employees with flexibility and professional freedom to perform their duties without direct supervision [5] and choose when and where to complete their work [6]. Another study on the feasibility of long-term remote work concluded that it offers employees greater flexibility, trust, and a better work-life balance, and significantly increases productivity by reducing commute times [7]. Remote work enables employees to choose their work hours to suit other aspects of their personal lives [8]. Increased personal time and independent schedules empower employees to better balance their work and personal lives, leading to positive work outcomes [8]. Remote work can reduce absenteeism by substituting telework with office presenteeism, thereby reducing travel stress, lengthening recovery from illness, and improving job satisfaction [9]. Scholars have also argued that job satisfaction remains relatively unaffected by working remotely [10].

However, we argue that remote work is associated with increased work-to-home conflict [11–13] and is likely to impact actual productivity. Several studies have argued that working remotely can blur the boundaries between home and work, leading to a disproportionate allocation of time and energy to domestic duties rather than professional ones [13,14]. This can prevent employees from meeting their work demands [15] and complicate their ability to balance their home and work roles, thereby increasing home-to-work conflict [14,16].

In this paper, we attempt to address the following questions:

- How prepared are people to accept remote work as their new environment?
- What impact will remote work have on employee productivity?

Now, since hybridization has been accepted as a new work format [17], a study on productivity will add significance to the existing quest for the impact of technology in the workplace and the continuation of remote and hybrid work models as an alternative to the regular workplace known to employees since the Industrial Revolution. Our insights into the potential challenges and benefits of remote work will help provide a more nuanced understanding of its value across contexts. Our paper fills a gap in the literature by focusing on workplace evolution during and after the pandemic, specifically in a rapidly developing country like India. This is an important area of study, as the pandemic has accelerated changes in work practices and organizational dynamics worldwide and has unique implications for countries, such as India, that are undergoing rapid economic and technological transformations. The remainder of this paper is organized as follows: Section 2 reviews the literature; the model and hypothesis development are deliberated in Section 3; and Sections 4 and 5 present the research methodology and analysis. The analysis results are discussed in Section 6, and the conclusion is presented in Section 7.

## 2. Theory and literature review

Fundamentally, work productivity is the value produced per unit of the cost required to do so. Therefore, any increase in individual productivity will increase organisational productivity [18]. Within the context of the knowledge economy, the growth in remote work that occurred even before the pandemic indicates an economic shift, placing greater emphasis on the knowledge worker, who generates value outside the confines of the office [19]. Hence, remote work is compelling organisations to provide increased temporal flexibility. Knowledge work is presumed to be nonlinear and requires around-the-clock engagement [20], making formal working hours redundant in a highly connected world. We based our study on three theoretical premises. First, we based our argument on the flexible firm model proposed by [21–23], which suggests that not all workers perform equally during traditional work hours. This supports the idea that knowledge work is nonlinear and may require around-the-clock engagement. This flexibility enables workers to better align their work schedules with their individual productivity peaks, ultimately improving efficiency and effectiveness [20].

We also used the theory of organisational adaptation [24] to support our argument. The theory states a “need for employers to revise employment practices to adapt to changing circumstances”. Chakravarthy [25] stated that adaptation is one of the most popular concepts in organisation theory and strategic management. By detaching work from a physical office space, organisations can leverage technology to create environments where work integrates seamlessly with it. This adaptation provides temporal flexibility, allowing the organisation to attract and retain workers who do not strongly identify with the office location or organisational structure [26]. Remote work is more appealing to workers who seek greater freedom and work–life balance because they do not need to be physically present.

The third premise of our argument relies on the conservation of resources theory, which argues that individuals and organizations strive to minimize the loss of resources, such as time, affect, and cognitive resources, by gaining other resources to achieve work goals [27]. We are applying the conservation of resources theory to develop an understanding of the impact of remote work on the work environment and on perceptions of work productivity and the meaning of work. COR theory emphasizes the importance of remote working and hybrid work models in shaping the future of the workplace, rather than advocating a return to past practices. Since employees have experienced increased autonomy and flexibility in remote work, we argue that productivity will remain unaffected, given the significant resources gained during the COVID-19 pandemic [28]. We also expect this theory to help us understand the phenomenon of stress in a rapidly changing workplace context. We further contend that remote work would require further commitment and investments in the interest of an evolving workplace [29]. Also, a study by Bloom et al. [30] in the context of Chinese technology company found that hybrid working improved job satisfaction and reduced quit rates by one-third.

Building on the above theories, we propose that remote work will impact employee productivity and the workplace in the near and long term. It will also encourage employees to learn new skills, optimise their time, rethink their interpersonal relationships, and handle stress to adapt to an ever-changing workplace. We also speculate that the lessons learnt from COVID-19 will have a lasting impact on how knowledge workers adapt to a new and unfamiliar working environment.

### 2.1. Remote work

Over the decades, remote work has evolved from simply working from home to a sophisticated and widely accepted form of flexible work that leverages technology to enable employees to work from virtually anywhere. Weerasinghe and Jayawardana [31] observed that remote work enables employees to better balance their work and personal lives. This balance contributes to improved employee motivation and morale. Mustajab et al. [32] found that remote work enables employees to schedule their work flexibly, which increases job satisfaction. Having control over one’s work schedule can enhance productivity and well-being. Magnusson [33] noted that remote work allows employees more time with their families. This benefit is especially significant during times of crisis, such as the pandemic, as it fosters stronger familial relationships. Senik et al. [34] highlighted the dual advantage of working remotely during the pandemic: employees could spend more time with their families while reducing their risk of COVID-19 infection.

### 2.2. Employee productivity

Productivity directly impacts on a company’s bottom line, making it a significant factor in organizational success. Indeed, scholars such as McPhail et al. [18] have recently highlighted this point. A highly productive workforce can increase output, profitability, and competitiveness. Consistent with self-determination theory, recent studies have suggested that employees become more productive when they are offered autonomy [35]. Hence, we argue that remote working affords greater autonomy and offers the opportunity to reduce the adverse impact of work-home conflict, thereby increasing work motivation and productivity.

Other studies have also argued that remote work benefits both employees and organisations, as it can reduce costs [36], motivate employees, and offer them greater work–life balance [18]. For example, organisations can use remote work to control their infrastructure, water, electricity, phone, and Internet costs, as well as cleaning and security costs. Other studies have argued that remote work promotes planning skills [37] and employee satisfaction [38].

### 2.3. Skill development

The concept of skill development is dynamic and multifaceted [39]. Skill development is not a constant process but a continuous and context-dependent one that depends on individual goals, organisational requirements, and external environmental changes. Crisis exacerbates the need for skill upgradation, particularly for knowledge workers. We base our argument on the transformational learning theory, which posits that individuals strive to learn during a crisis or through deep self-reflection [40]. We argue that COVID-19 brought about a significant crisis in employees’ work lives and left them anxious about their current skills. Other studies during the COVID-19 pandemic also argued that the crisis and rapidly changing employment market drove rapid skill acquisition [41]. Our argument that skill acquisition will take priority in an evolving remote-work scenario is supported by theories of skill development and lifelong learning. In addition, scholars from the Human Capital School have encouraged organisations to invest in employee skills to help organisations achieve their strategic objectives [42]. As Laleman et al. [43] argued, such skills evolution includes technical, social, emotional, and physical skills required to demonstrate good workplace behaviour. Developing skills is a lifelong process; people must constantly improve and learn.

### 2.4. Time availability

Scholars have agreed that time availability and work productivity have a non-linear relationship. However, some studies argue that time availability is an important factor influencing employees’ attitudes towards work. Studies have shown that as time pressure increases, work productivity decreases [44]. Braun [45] provided a comprehensive overview of studies examining the link between time perception in a work context and one’s mental well-being. Time-related phenomena, such as insufficient time and deadline pressure, are common stressors that can impact job satisfaction, health, and performance. Consistent with Job Demand Resources Theory, productivity suffers when work exceeds available time [46]. Studies during the pandemic show that net time availability increased [47]. Hwang [48] examined the effects of work pace and time pressure on performance. However, scholars have argued that there is a non-linear relationship between time availability and work productivity. In his pioneering work, Parkinson [49] argued that the availability of excess time often expands the work. Similarly, in a recent empirical study, Yuan, Y. et al. [50] proposed that work efficiency decreases following a rise in productivity due to a positive nonlinear mediating effect of learning behavior. In addition, findings from Kniffin et al. [51] during the COVID-19 pandemic show that remote work blurs the work/home boundary and is associated with lower work excitement and greater procrastination.

Finally, time availability also determines how engaged and committed employees are. Employees with sufficient time to complete tasks and manage their workload may exhibit more positive attitudes and behaviours at work.

### 2.5. Interpersonal relations

Interpersonal relations are connections between individuals characterised by reciprocal behaviours and favourable connections. These relationships can occur in various settings, including personal and professional environments, and involve mutual interactions that benefit both parties [52]. Literature on self-determination theory and social presence theory strongly argues that interpersonal relations affect employees’ work productivity. Notably, social presence theory posits that, in computer-mediated interpersonal relationships, co-workers’ presence significantly affects the quality and outcomes of interpersonal communication [53]. In remote work, social presence is significantly reduced. This can result in miscommunication, weakened team bonding, misunderstandings, a differentiated approach to achieving organizational goals, and reduced productivity. Evidence suggests that poor social connections can significantly hinder workflow and reduce team efficiency [53]. Successful interpersonal relationships often develop when individuals understand, care for, and support one another. Enduring interpersonal relationships play a crucial role in achieving organisational goals. Interpersonal relations are essential for fostering mutual support and achieving personal and professional goals.

### 2.6. Stress

Organisational or situational stress influences the work environment and organisational actions, determining employees’ job satisfaction and productivity [54]. Stress and adaptation theory suggests that high-magnitude crises can significantly impact productivity. Stress can work both ways: in some cases, it can enhance productivity, while in others, it can reduce it, depending on how individuals and organizations respond to and adapt to emerging complexities [55]. Empirical evidence suggests that individuals experience swift adaptation during and after a high-magnitude crisis by accepting ‘challenge stress’ and maintaining focus on work productivity [56]. In addition, ambiguity and organisational stress can harm employees’ work attitudes, job satisfaction, and productivity [54]. Therefore, individuals and Organizations must monitor stress and its impact on employee well-being and performance in remote work settings.

## 3. Model and hypothesis development

**Fig 1:**
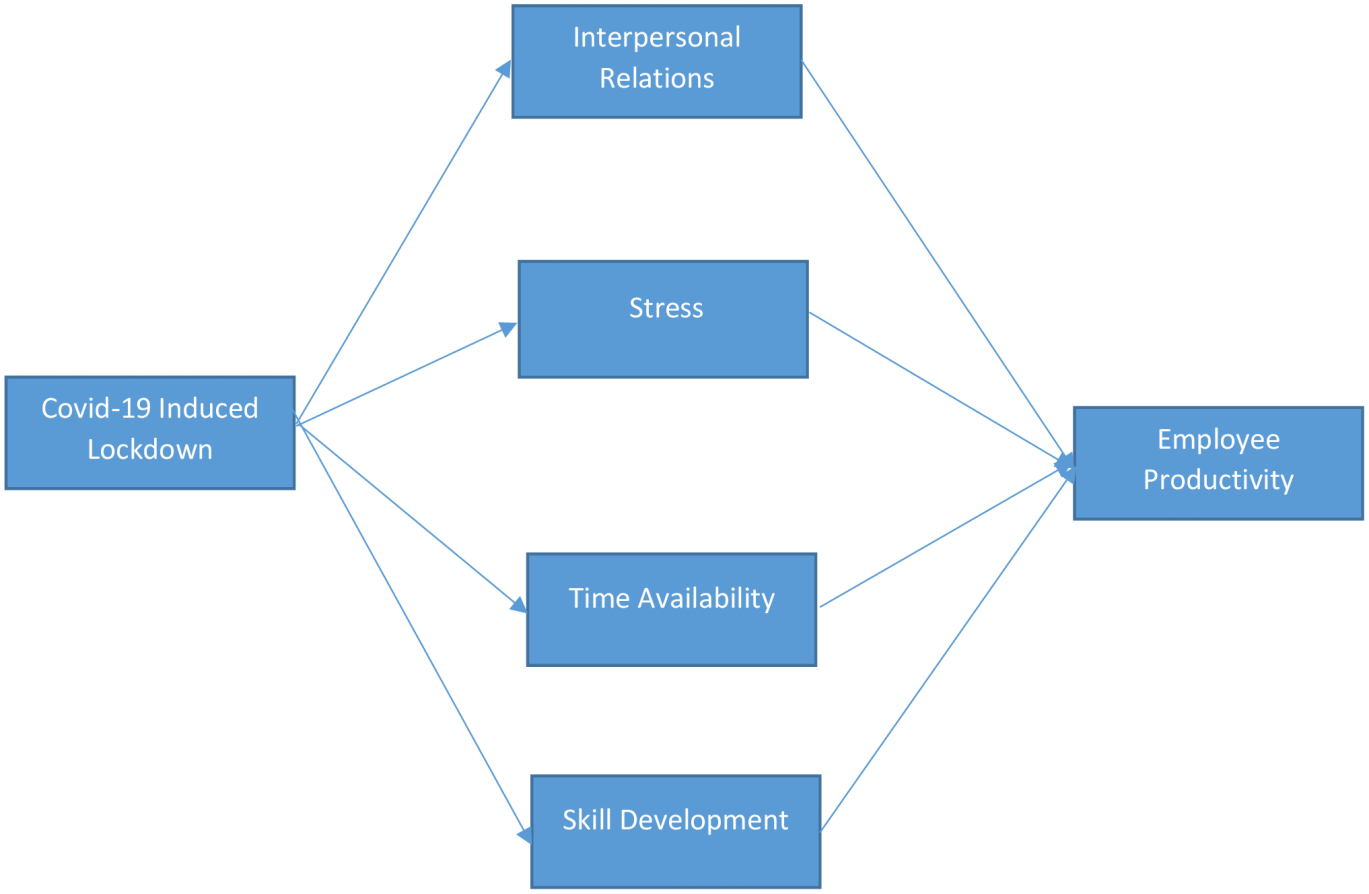
The conceptual model.

Derived from the theories discussed in Section 2, the following model outlines the interplay among the factors shaping employee productivity (see Fig 1). These sections also introduce our hypotheses.

### 3.1. Interpersonal relations and employee productivity

A deep density of interpersonal relations fosters a collaborative and supportive work environment, leading to greater procedural efficiency and overall organisational productivity [57]. Positive interpersonal workplace relationships indirectly influence customer satisfaction by improving service quality and employee customer support. Strong workplace friendships lead to higher organisational commitment and employee engagement, which in turn improve performance and dedication to organisational goals [58]. In a study on building work engagement in organizations, Simbula et al. [59] confirmed that social support has a positive impact on work engagement when respondents are approached twice, with a three-month lag.

Hypothesis 1 posits that positive interpersonal relationships enhance organisational effectiveness and employee well-being.

H1: There shall be a significant relationship between interpersonal relations and employee productivity during COVID-19-induced remote working.

### 3.2. Stress and employee productivity

Richardson [60] highlighted that employees’ mental health significantly determines their productivity. When employees experience stress or mental health issues, their ability to focus, make decisions, and perform their roles effectively may be hindered. Pinheiro et al [61] emphasized the adverse effects of stress on employees’ decision-making ability, performance, and behaviour. High stress levels can lead to low performance, increased turnover, absenteeism, and higher employee expenses. Finally, stress affects employees’ quality of life [62]. Serenko [54] cited empirical research supporting this statement, noting that a large proportion of Chinese workers suffer from work-related stress that harms their performance and mental health. According to Chao et al. [63], in a study of healthcare workers in rural Taiwan, work stress was found to positively influence job performance. Laguna et al. [64] claimed that employees who experience high levels of work stress are prone to mental illness and negative behaviour patterns such as anxiety, aggression, and isolation. Marsh et al [65] emphasize the need for organizations to address the mental and physical health ramifications of the dark side of digital working. In conclusion, the attached literature highlights the role of workplace stress and its impact on work performance and employees’ mental health.

H2: A significant relationship exists between employee stress and productivity during the COVID–19 remote working.

### 3.3. Time availability and employee productivity

A complex relationship exists between the time one spends working and their productivity. Bachrach et al. [66] suggested that time can be used to measure work and employee productivity. In an earlier study, Tatom [67] argued that researchers have historically assumed that the only way to increase the amount of work a person produces is to require them to work more hours. Some studies, such as Ilmakunnas [68], have shown that overtime work is positively associated with work output. This indicates that the more time one spends working, the higher their productivity level. However, more recent studies, including Tatom [67] and Shepard and Clifton [69], have shown that working time can diminish work productivity. This indicates that the relationship between time and productivity is not linear or straightforward. Dolton et al. [70] and Lu and Lu [71] further supported this assertion, suggesting that increased work hours lead to poor work quality and quantity.

H3: A significant relationship exists between available work time and employee productivity during COVID–19 remote working.

### 3.4. Skill development and employee productivity

Skill development can be defined as a long-term, strategic effort aimed at achieving a specific goal, initiated by either the learner or their employer. Such programs aim to improve workplace productivity by acquiring or refining skills. Tansky and Cohen [72] claimed that investments in workplace skills development improve employee efficiency and enhance one’s work-life balance. According to Runhaar et al. [73], investing in skill development means investing in employees’ future work lives. This is a long-term investment that keeps employees deployable and continually challenges them. The authors further asserted that individuals must identify the capabilities that need to be developed and then deploy available resources to ensure they remain competitive in the labor market. In a comparative analysis of workforce efficiency, customer engagement, and risk management strategies in Nigeria and the United States, Egieya et al. [74] discuss the importance of workforce efficiency for organizational success. Joshi et al. [75] found that acquiring new work skills can encourage employees to become more engaged and contribute more to their workplace. To propose an alternative conceptual approach to the HRM framework, Pathomphatthaphan et al. [76] presents a model that highlights how the framework is designed to suit each stage of an employee’s life cycle. Also, Lakshmi Devi et al. [77] in a study on AI-based skill development training for employability discussed the importance of critical thinking, advanced AI technology, communication, and interpersonal skills. This evidence suggests that both skill development and deterioration are important from the perspective of both the individual and the organisation. Overall, literature underscores the importance of skill development for individual career growth and organisational success.

H4: Employees can significantly improve their professional skills during the COVID–19–induced remote work period.

**Fig 2:**
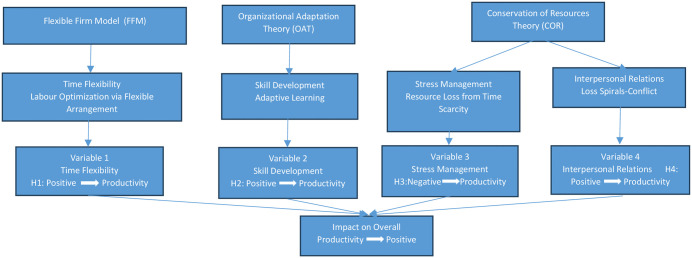
Theoretical integration logic diagram.

The proposed model with theoretical integration is presented (see Fig 2).

## 4. Research methodology

### 4.1. Research design

A research design reflects the strategy adopted by the researcher to coherently and logically integrate study components to address the research problem. A research design is a strategic framework for action that bridges the research questions and the execution of the research [78]. For the present study, a descriptive research design was adopted to identify factors affecting the productivity of Indian employees working from home during the COVID-19 pandemic. Descriptive research is a research method that attempts to provide an accurate description of existing phenomena. A phenomenological study typically expects the researcher to develop meaningful research questions that relate to the individual’s lived experience [79]. Data were collected during the COVID-19 pandemic from employees working at public and private organisations in India.

### 4.2. Sample and data collection

Productivity was measured using a questionnaire developed by Koopmans et al. [80], with items modified to suit the ongoing pandemic. Online survey platforms were used to collect data from employees working from home. Of the 1,850 employees approached, we received usable responses from 646, representing a 35% response rate. The data cleaning process involved removing invalid and straight-line responses. Missing values were handled using the mean substitution method [82]. A test for nonresponse bias was conducted by comparing early and late responses. We concluded that a nonresponse bias did not impact the data, as all differences were statistically nonsignificant.

The sample consisted of 71.4% male and 28.6% female respondents. Respondents in the 30–40-year-old age group formed the majority of the sample (71.2%). Most worked in the service industry (86.7%) and the private sector (93.2%). The majority (63.5%) were mid-level employees, while the remainder held junior and senior-level roles. Most respondents (64.5%) worked remotely for more than six months. The majority (50.2%) were married, and 66.4% had between two and 10 years of work experience.

### 4.3. Survey instrument

Employee productivity was measured using a questionnaire developed based on Koopmans et al. [80] and the extant literature. The instrument items were revised to accommodate the study constructs without altering the instrument’s basic framework. An existing instrument was modified because no survey instruments were designed and validated during a similar pandemic. Twenty-three items assessed employee productivity on a 7-point Likert scale, where one indicated “strongly agree” and seven indicated “strongly disagree”. The questionnaire included questions regarding respondents’ demographic profiles. Face validity was established by removing ambiguity from the wording of questions. Some negative-direction survey items were reverse-coded to improve response objectivity. The questionnaire was further designed to account for participants’ ability to understand and answer the questions. Example survey items include, “I could carry out my work efficiently during COVID-19, “I could not contribute enough to my work during COVID-19,” and “I took on challenging tasks during COVID-19.”

Harman’s single-factor test was conducted to address common method bias. The test extracted multiple factors from factor analysis, with more than one eigenvalue. The results indicated that a single factor could not account for the covariance of most variables [81]. Therefore, standard-method bias was not a concern in this study. To assess the questionnaire’s reliability, Cronbach’s alpha was estimated and found to be 0.783. The items displayed high internal consistency, well above the 0.6 threshold [82].

## 5. Data analysis

### 5.1. Measurement model evaluations

Two-stage SEM was used to analyse the structural and measurement models, as it reduces the influence of their interactions [83]. In the first stage, confirmatory factor analysis (CFA) was applied to assess the measurement model. This involved evaluating the unidimensional and causal relationships between items and constructs. Construct reliability and validity were examined before the second stage. In the second stage, the structural model was examined to assess the relationship between exogenous variables (i.e., time availability, interpersonal relations, stress, and skill development) and endogenous variables (i.e., employee productivity). Multiple fit indices were examined [84] to assess whether the selected variables were adequate for the study. The following fit indices are reported in Table 1: chi-square (χ2), the ratio of chi-square values to the degrees of freedom (χ2/df), the goodness of fit index (GFI), the comparative fit index (CFI), and the root mean square error of approximation (RMSEA).

**Table 1:** Confirmatory factor analysis results.

	χ2	df	χ2/df	GFI	CFI	RMSEA
Model	673.523	214	3.147	0.916	0.902	0.058

Source: Extracted by authors

#### 5.1.1. Measurement model testing.

**Fig 3:**
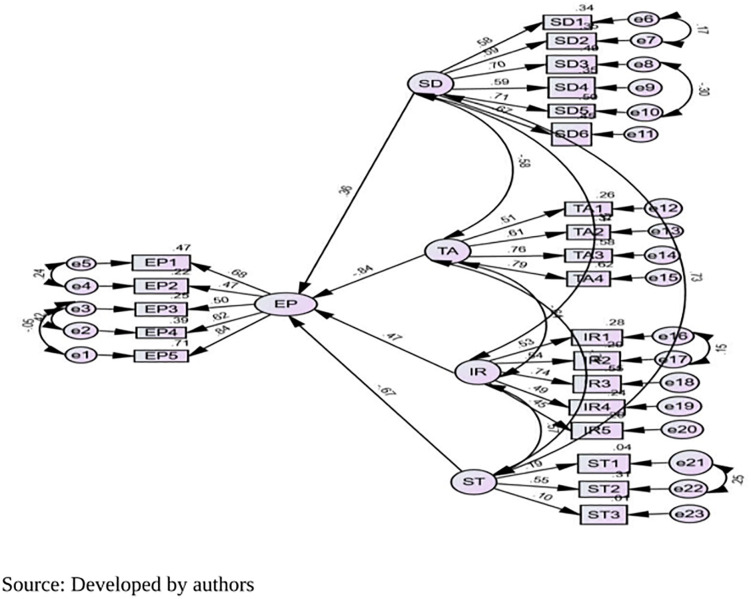
Structural equation model.

The measurement model describes how each measure is loaded on a specific variable [83]. The measurement model was examined by incorporating all variables: time availability, interpersonal relations, stress, skill development, and employee productivity. CFA was performed to assess model fit. IBM SPSS AMOS 27.0 and the maximum likelihood method were used to estimate the model parameters (see Fig 3). However, employee productivity was first tested for normality using Mardia’s multivariate kurtosis and a 0.05 significance level; the critical ratio was less than 1.96, indicating that the measure was normal. The results suggested an acceptable fit, with χ² = 673.523 (df = 214, p = 0.00), a χ²/df ratio of 3.147, a GFI of 0.916, a CFI of 0.902, and an RMSEA of 0.058 [39].

#### 5.1.2. Instrument reliability and validity.

To assess the convergent validity of each construct, we examined the parameter estimates and their associated t-statistics [85]. Evidence of convergent validity was observed, as all indicator loadings (λ) were statistically significant [86]. In this study, all items considered demonstrated significance. A scale purification process was then performed, and indicator variables with standardised factor loadings smaller than 0.4 were removed [86]. This process helped ensure the scale’s quality. To further evaluate the convergent validity of each construct, we calculated composite reliability and the average variance extracted (AVE). These measures provide insight into how well each construct is captured by its indicators. We also conducted tests to detect potential multicollinearity. The low variance inflation factors for constructs suggested that no potential bias arose from correlated formative indicators. Table 2 presents the final results of the measurement model. Composite reliability, which represents the shared variance among indicators measuring an underlying construct [87], exceeded the threshold of 0.6. The AVE for each construct, except interpersonal relations, exceeded 0.4. According to Fornell et al. [87], the acceptable threshold for AVE is greater than 0.4 on most reflective scales. Numerous published studies include one or more reflective scales with AVEs below 0.4 [88]. Therefore, our constructs can be considered reliable and unidimensional [85,89].

**Table 2:** Construct- items measures.

Construct	Items	Item Code	Standardized Loadings	AVE	CR
**Employee Productivity (EP)**	During the lockdown, I feel good about my professional productivity	EP1	0.682		
	I could not contribute enough to my work during the lockdown	EP2	0.472	0.407	0.710
	I never lost sight of my professional responsibility	EP3	0.502		
	I never lost sight of my Goals during the lockdown period	EP4	0.622		
	I can carry out my work efficiently during the lockdown	EP5	0.841		
					
**Skill Development (SD)**	On my initiative, I started new tasks when my old tasks were completed during the lockdown	SD1	0.584	0.412	0.807
	I took on challenging tasks during the lockdown	SD2	0.588		
	I worked on keeping my job-related knowledge up-to-date	SD3	0.698		
	I came up with creative solutions for new problems during the isolation of lockdown	SD4	0.589		
	I took on extra responsibilities	SD5	0.706		
	I continually sought new challenges in my work	SD6	0.671		
					
**Interpersonal** **Relations (IR)**	The lockdown has not impacted my professional relationships	IR1	0.528	0.319	0.730
	My boss and colleagues were very supportive during the lockdown and were available for me	IR2	0.538		
	I could adequately participate in work and interact with my colleagues during the lockdown	IR3	0.740		
	The future of my organization is looking brighter after the lockdown.	IR4	0.488		
	I actively participate in all online meetings	IR5	0.452		
					
**Time Availability (TA)**	During the lockdown, I lost track of my priorities	TA1	0.507	0.458	0.692
	During the lockdown, I could maintain a good balance of life, and it is improving	TA2	0.608		
	I can plan my work so that I can finish all the tasks during the lockdown on time	TA3	0.763		
	I managed my time well during the lockdown	TA4	0.789		
					
**Stress (ST)**	The lockdown has made me anxious to develop my knowledge and skills	ST1	0.712	0.421	0.683
	My professional life will never be the same after the lockdown	ST2	0.544		
	Lockdown denied me the opportunity to learn and grow professionally	ST3	0.678		

Source: Extracted by authors from their analysis

To assess discriminant validity, we compared the AVE of each construct with the square of the construct correlations that exist with other latent constructs. All the AVEs were greater than the correlation squares. These results suggest good discriminant validity of the constructs [87].

Robustness Tests

To verify the established model’s results, a robustness test assessed consistency across males and females. The results are reported in Table 3. The resulting indices, like x2, a x2/df, a Goodness of Fit Index (GFI), a Comparative Fit Index(CFI), and a Root Mean Square of Approximation, suggested an acceptable fit for both males and females.

**Table 3:** Robustness test results.

Hypothesis/Path	Full	Males	Females
H1A: IR ^→^ EP	0.47^***^	0.537^*^	0.529^***^
H2A: ST ^→^ EP	−0.67^*^	−1.022	−0.613
H3A:TA ^→^ EP	−0.84^***^	−0.869^**^	−0.771^**^
H4A:SD ^→^ EP	.0.36^**^	0.669^*^	0.250

***p < .001. **p < .01. *p < .05. Female sample size:185

Source: Extracted by authors from their analysis

From the above table, it can be inferred that the impact of interpersonal relationships on employee productivity is significant for both genders; however, it is slightly greater for males than for females. Similarly, time availability significantly affected employee productivity for both genders; however, the effect was slightly greater for females than for males. Interestingly, the results indicate that stress has a significant impact on employee productivity when gender responses are taken together but becomes insignificant when considered separately. Finally, the impact of skill development on employee productivity is significant for males and insignificant for females.

The differences observed between the full-sample and gender-specific models suggest potential heterogeneity in the effects of remote work on employees. The full model reflects pooled average effects, while sub-sample regressions capture group-specific dynamics. Variations in statistical significance may also stem from a smaller sample size in the female subgroup, resulting in lower statistical power.

### 5.2. Hypothesis testing

To examine the hypothesized relationships, a two-step approach of confirmatory factor analysis (CFA) and structural equation modeling (SEM) was adopted [82] using AMOS 27.0. Table 4 shows that interpersonal relations (β = 0.47, p < 0.001) and skills development (β = 0.36, p < 0.01) had a positive impact on employee productivity. It also shows that stress (β = −0.67, p < 0.05) and time availability (β = −0.84, p < 0.001) had significant adverse effects on employee productivity among pandemic-induced remote workers. Therefore, hypotheses 1–4 are accepted.

**Table 4:** Hypotheses and structural paths.

Hypothesis	Path Relationship	β	SE	Results
H1	IR ^→^ EP	0.47^***^	0.14	Supported
H2	ST ^→^ EP	−0.67^*^	1.18	Supported
H3	TA ^→^ EP	−0.84^***^	0.19	Supported
H4	SD ^→^ EP	.0.36^**^	0.19	Supported

***p < .001. **p < .01. *p < .05.

Source: Extracted by authors from their analysis

## 6. Discussion

While the literature on this topic remains nascent due to the pandemic’s sudden onset, research has begun to explore aspects of the people–technology interface and its impact on workplace productivity. McPhail et al. [18] highlighted the scarcity of literature in this area and emphasised the need for further research. They noted that many existing studies have prioritised investigating individuals’ well-being over other factors, potentially overlooking broader implications for workplace dynamics and productivity [90]. Drawing from the influential work of [91] that theories ought to move beyond the realm of variables and data and must attempt to explain “why” relationships occur. This work adds to the existing body of work on how remote work impacts productivity through a set of interrelated and significant social, technical and behavioral mechanisms emerging from an extraordinary crisis.

To address this research gap, our study aimed to answer several important questions. Using a sample of 646 participants, we first sought to determine whether the initial productivity gains from working remotely are sustainable. Based on our findings, we argue that the COVID-19-induced remote-workplace revolution has the potential to serve as a long-term engine of productivity and motivation.

Our findings align with existing research on the relationship between people and productivity. Such studies emphasized the important role that technological infrastructure plays in driving productivity levels in an organizational setting. By providing a multidimensional perspective on the nature of remote workers’ people–technology interactions, our model suggests that the following four constructs significantly impact workplace productivity: skill development, interpersonal relations, time availability, and stress. Specifically, we observed that remote work facilitated skill development by providing employees with opportunities for self-directed learning and requiring them to adapt to new technologies and work processes. This phenomenon aligns with recent perspectives on adaptive capability and workforce flexibility, wherein employee productivity drives organizational growth. Remote work influenced interpersonal relationships through virtual communication tools, which played a crucial role in maintaining connectivity among team members. Remote work also influenced employees’ perceptions of time availability, as the flexibility it afforded enabled individuals to manage their schedules more effectively and allocate time more efficiently. Furthermore, our findings indicate that stress and working from home are not significantly related. Overall, our findings underscore the complex interplay between remote work and employee productivity, highlighting the importance of considering additional factors beyond physical location. By illustrating how remote work can affect employee productivity, our study provides valuable insights for organisations seeking to optimise their remote work policies and practices in the post-pandemic era.

The study on family–work conflict and stress-induced work have several key implications regarding the impact of remote work, particularly during the COVID-19 pandemic. Our findings suggest that employees demonstrate considerable resilience in managing the crisis. Contrary to previous findings, the results indicate a positive sentiment toward moving from physical to digital. Contrary to Duxbury’s [92] findings, this study indicates that remote work has an insignificant impact on work-family conflict. Duxbury’s work indicated an increased work-family conflict due to the dissolution of physical and temporal boundaries between roles. Our findings indicate no such friction in family and work roles. Our findings are consistent with those of Kniffin et al. [51], who found that technological evolution and rapid adaptation reduce work-family friction. Also, other influential studies [93] argue that remote work has become a new workplace reality, and families have recalibrated their work and family expectations in favour of remote work. Going back to full physical office days can prove counterproductive; scholars favour a hybrid work arrangement as the future of work. Furthermore, our findings indicate that stress and working from home are not significantly related. This runs contrary to our expectations. This suggests that remote work provides a buffer against primary sources of stress, as described by the job demands–resources model [94]. While remote work challenges employees’ time management skills, it also allows them to manage their physical and mental recovery from pandemic-induced stress. Autonomy in setting one’s schedule is positively related to productivity and work engagement. Ultimately, developing practical time-management skills is crucial for remote work to succeed.

From a Conservation of Resources (COR) perspective, availability of time should not be construed as net gain of time resource. Even if temporal flexibility may assist work autonomy, however, more or less time availability can also lead to workplace imbalances, particularly deficit in attention can lead to resource depletion or poor work productivity. Another, fallout can be conflicting work and personal responsibilities. The disproportionate attention can increase cognitive effort to reach productivity targets. The above adds to the COR theory by arguing that time is a dual-condition resource. In one hand effective use can enhance productivity and in the other hand poor utilization can trigger resource loss undermining the productivity and employee well-being.

We also found that individual development was crucial in driving workforce changes during the COVID-19 pandemic. Employees realised they needed to expand their skills to align their abilities with shifting business trends and demands. Additionally, it implies that the work mode will shift to a new normal, characterized by more refined work processes that support choice and increased resources, allowing employees to use their specific skills in the virtual work environment. In summary, extending remote work opportunities beyond the COVID-19 pandemic may benefit organisations and employees by reducing stress, increasing productivity, and improving work–life balance.

### 6.1. Theoretical contributions

Our results provide insight into the substantial impact of a crisis on employees’ views of their work. We argue that this work theoretically contributes in three ways. Consistent with the work of [95] this study enhances the existing premise of remote work productivity by highlighting mechanisms through which remote work affects employee outcomes. Particularly, by specifying the role of relational, developmental, temporal and psychological aspects in shaping the perception of work productivity.

Secondly, supporting [91] argument that theories should link constructs and logic meaningfully. This work contributes to the job demands-resources theory from a socio-technical perspective mediated by remote work systems. Hence, contributing to the understanding of the dynamic interplay of employee resource mobilization and technology driven work arrangement. This is likely to strengthen our understanding of remote work effectiveness and the rise of digital workplaces.

Third, this study employs efforts to contextualize a global workplace topic. Much of scholarly studies on remote work has a western context and western organizational setting. This study is an attempt to extend the argument of Homer & Lim’s, [96] that theories ought to be contextually grounded in a vastly interconnected world. By studying Indian employees during an extraordinary crisis demonstrates contextual conditions in an evolving workplace.

Finally, this work supports the flexible firm theory that remote work is not just restricted to temporary and contractual workforce, it is spans across all forms of employment, prompting organizations to integrate technology, human resources and employee well-being.

They suggest that employee well-being and work–life balance must be considered in a professional environment. During the COVID-19 crisis, employees were forced to reassess their professional futures, sparking an increased curiosity about new working methods. The current study’s results align with existing evidence and contribute to a theoretical understanding of remote work productivity, employee engagement, and professional stress. Additionally, our results align with flexible firm theory, suggesting that organisations must be adaptable and flexible in the face of social change.

Our results also suggest future directions for researchers examining various aspects of remote work, including gender differences in responses to remote work, industry-specific adaptations to remote work, team dynamics in remote work environments, and the impact of digital technologies on the evolution of the work environment. Moreover, it may be worthwhile to investigate virtual work opportunities as a strategy for employee attraction and retention [51].

### 6.2. Practical implications

This study has practical implications for managers in the post-COVID-19 era. Our findings may help leaders rethink their conceptions of the conventional workplace, encouraging them to view remote and hybrid working conditions as a human resources (HR) advantage. They may view such options as motivation, attraction, retention, and productivity management tools. With the world’s growing population and on-the-ground congestion, remote work seems destined to become a common employee preference. This is particularly true in India, where workers face pressing issues such as pollution and heavy traffic, which are reducing their overall health and initiative to perform. Remote work alleviates some of these stressors while promoting work–life balance and flexibility. Therefore, the responsibility for adaptation rests with the institution and the individual.

The results of this work would be of tremendous value to HR managers, who could utilize our findings to develop effective training programs and engagement models that help employees thrive in virtual workspaces. Our findings may also help HR managers develop initiatives to enhance digital literacy, promote team collaboration in virtual conference spaces, and foster a virtual-friendly culture of employee well-being. HR managers may also develop a comprehensive virtual workforce plan to maintain a thriving work environment. The pandemic has forced companies to work digitally. As a result, HR managers must explore opportunities to implement systems that help their enterprises take full advantage of the vast options available globally in the digital world. These systems, processes, and models should be designed to fully utilise the possible digital benefits for organisational productivity, collaboration, and innovation. Additionally, HR managers can utilize the findings of this research to address the critical issue of employee retention. HR managers can use remote work as a strategy to reduce employee attrition. The findings of this study support recent research indicating that remote working is well-endorsed by knowledge workers, with reports suggesting it has already reduced attrition by 35%. [97], without negatively impacting productivity, performance, teamwork, and promotion opportunities [4]. According to the above organizations, such as Meta, Apple, HSBC, Volkswagen AG, Bupa, and Citi Standard Chartered Bank, they have all publicly admitted plans to make remote and hybrid working a new workplace arrangement [98]. We also believe that this study’s findings support the United Nations’ (UN) sustainability goals. Particularly, commitments to decent work (SDG8), gender equality (SDG5), and good health and well-being (SDG3) are emphasized, as they provide each employee with an equal opportunity to utilize remote and hybrid working options to balance their work and life [99–100]. If achieved, this can significantly contribute to the overall well-being of families and society.

### 6.3. Limitations

The limitations of the present study reveal avenues for further research. This study suffers from some limitations, first, time availability and stress were measured as unidimensional constructs, which may not fully capture the complexity of employees’ remote work experiences. Particularly the measurement approach did not capture the distinction between discretionary time availability and work autonomy. This can limit the understanding of non-linear impact of time availability on employee productivity. Future studies can cover by adopting multidimensional measures for measuring the impact of flexibility and its impact on productivity.

Similarly, stress as a construct excluded various sources of stress in remote work environments. For instance sources such as digital exhaust, emotional exhaustion, social isolation etc. was not covered in the study. As a result, the significance of results should be cautiously interpreted. Other sources stress can have a significant impact on well-being and work productivity.

Future researchers should conduct longitudinal studies examining the shifts in attitudes, behaviours, and outcomes related to remote work. By integrating data from before, during, and after the pandemic, scholars may better understand the long-term effects of remote work on productivity, well-being, and organisational measures. Similarly, future research could investigate sector-specific implications of remote work, revealing which industries are most likely to continue implementing or offering remote work in the post-pandemic period. Such findings could inform the development of implementation strategies and policies related to remote work. Another limitation of this study is the non-generalizability of its results to manufacturing and other sectors. Representation from manufacturing and public sector organizations is disproportionate. Hence, the findings reflect the impact of remote work within the private sector services industry. Going forward, scholars can conduct studies on manufacturing and public-sector enterprises to enhance generalizability. This will help in establishing sector-specific dynamics.

Research with a more robust and diverse sample could enhance the generalizability and statistical power of the findings, thereby providing more convincing evidence for the results presented here. Additionally, further research could examine the mediating and moderating roles of engagement, satisfaction, and motivation in productivity across different working conditions.

Another important issue for future research to address is digital exhaust and its impact on work. This may include studying how digital excess, information overload, and digital distractions influence the well-being and performance of workers in a digitally remote, accessible, and practical work system. Overall, research into these areas could lead to a more nuanced understanding of the opportunities and challenges of remote work, ultimately helping build more transformative and inclusive work policies.

## 7. Conclusion

The changes to the working environment in the post-COVID-19 era have had positive and negative effects. Regarding the positive effects, both employers and employees have accepted the shift from in-office to remote work as a viable alternative to the physical workplace. On the negative side, it is too soon to conclude the future of remote work post-COVID-19.

Earlier in the study, we questioned employees’ preparedness to accept the emerging new work environment. However, we can assume that socially focused and productivity-based issues will remain central to the workplace. The present analysis suggests that providing employees with greater flexibility, such as remote or hybrid work options, can enhance productivity and promote better work-life balance. Allowing flexibility for different workplace configurations advances work–life balance and overall well-being, and has become rapidly accepted. Simultaneously, the shift to remote work has sparked conversations about employee well-being that have been historically overlooked at the policy level. We believe that organisations are beginning to grasp the important role of the home environment in their mental and physical health and workplace motivation. In addition, the long-held belief that daily commute and physical presence are deterrents in attaining work-life balance will lose some relevance. We can expect harmony between work and life, with options to work remotely while staying productive. This belief can have positive social consequences, including reduced work-family conflict. We are also likely to witness lasting changes in the workplace debate. Organizations must realize the importance of providing employees with flexibility and result-oriented work systems. We are confident that employees are now better prepared than before to accept remote and hybrid work environments as their new workplace and are adjusting to remain productive without frequent visits to the office.

## Supporting information

S1 File Inclusivity-in-global-research-questionnaire. (DOCX)

## References

[1] Goldstein J. An architecture of optimism in a post-pandemic society. 2020. http://example.com

[2] Bloom N, Liang J, Roberts J, Ying ZJ. Does working from home work? Evidence from a Chinese Experiment *. The Quarterly Journal of Economics. 2014;130(1):165–218. doi: 10.1093/qje/qju032

[3] Wigert B, Agrawal S. Returning to the office: the current, preferred and future state of remote work. Washington DC. 2022.

[4] Executives feel the strain of leading in the ‘new normal’. 2022. https://futureforum.com/wp-content/uploads/2022/10/Future-Forum-Pulse-Report-Fall-2022.pdf

[5] Kossek EE, Thompson RJ. Workplace flexibility. The Oxford handbook of work and family. 2015.

[6] McCloskey DW. An examination of the boundary between work and home for knowledge workers. International Journal of Human Capital and Information Technology Professionals. 2018;9(3):25–41. doi: 10.4018/ijhcitp.2018070102

[7] Krasulja N, Vasiljevic-Blagojevic M, Radojevic I. Working from home as alternative for acheving work-life balance. Ekonomika. 2015;61(2):131–42. doi: 10.5937/ekonomika1502131k

[8] Tremblay DG, Genin É. The demand for telework of IT self-employed workers. The Journal of E-working. 2007;1(2):98–115.

[9] Johns G. Presenteeism in the workplace: a review and research agenda. J Organ Behavior. 2009;31(4):519–42. doi: 10.1002/job.630

[10] Chuang Y-T, Chiang H-L, Lin A-P. Information quality, work-family conflict, loneliness, and well-being in remote work settings. Computers in Human Behavior. 2024;154:108149. doi: 10.1016/j.chb.2024.108149

[11] Hammer LB, Cullen JC, Neal MB, Sinclair RR, Shafiro MV. The longitudinal effects of work-family conflict and positive spillover on depressive symptoms among dual-earner couples. J Occup Health Psychol. 2005;10(2):138–54. doi: 10.1037/1076-8998.10.2.138 15826224

[12] Hill EJ, Ferris M, Märtinson V. Does it matter where you work? A comparison of how three work venues (traditional office, virtual office, and home office) influence aspects of work and personal/family life. Journal of Vocational Behavior. 2003;63(2):220–41. doi: 10.1016/s0001-8791(03)00042-3

[13] Schieman S, Young M. Is there a downside to schedule control for the work-family interface? J Family Iss. 2010;31(10):1391–414. doi: 10.1177/0192513x10361866

[14] Duxbury LE, Higgins CA, Mills S. After-hours telecommuting and work-family conflict: a comparative analysis. Information Systems Research. 1992;3(2):173–90. doi: 10.1287/isre.3.2.173

[15] Voydanoff P. Consequences of boundary-spanning demands and resources for work-to-family conflict and perceived stress. J Occup Health Psychol. 2005;10(4):491–503. doi: 10.1037/1076-8998.10.4.491 16248695

[16] Golden TD, Veiga JF, Simsek Z. Telecommuting’s differential impact on work-family conflict: is there no place like home? J Appl Psychol. 2006;91(6):1340–50. doi: 10.1037/0021-9010.91.6.1340 17100488

[17] Lamovšek A, Cerne M, Popovič A, Salem Mohammed S, Trinchera L. The work design puzzle: untangling its relationship with work-life balance across different forms of work.

[18] McPhail R, Chan XW (Carys), May R, Wilkinson A. Post-COVID remote working and its impact on people, productivity, and the planet: an exploratory scoping review. The International Journal of Human Resource Management. 2023;35(1):154–82. doi: 10.1080/09585192.2023.2221385

[19] Wilson JP, Campbell L. Developing a knowledge management policy for ISO 9001: 2015. JKM. 2016;20(4):829–44. doi: 10.1108/jkm-11-2015-0472

[20] Hardill I, Green A. Remote working—altering the spatial contours of work and home in the new economy. New Technol Work Employ. 2003;18(3):212–22. doi: 10.1111/1468-005x.00122

[21] Atkinson J. Manpower strategies for flexible organisations. Personnel Management. 1984;16(8):28–31.

[22] Atkinson J. Flexibility, uncertainty and manpower management. 1985.

[23] Atkinson J. Flexibility or fragmentation-the labor-market of the united-kingdom in the 1980s. Cahiers economiques de bruxelles. 1987;113:163–205.

[24] Sarta A, Durand R, Vergne J-P. Organizational Adaptation. J Manage. 2021;47(1):43–75. doi: 10.1177/0149206320929088 33424060 PMC7736401

[25] Chakravarthy BS. Adaptation: a promising metaphor for strategic management. The Academy of Management Review. 1982;7(1):35. doi: 10.2307/257246

[26] Felstead A, Henseke G. Assessing the growth of remote working and its consequences for effort, well‐being and work‐life balance. New Technol Work Employ. 2017;32(3):195–212. doi: 10.1111/ntwe.12097

[27] Hobfoll SE, Halbesleben J, Neveu JP, Westman M. Conservation of resources in the organizational context: The reality of resources and their consequences. Ann Rev Organizational Psychol Organizational Behav. 2018;5:103–28.

[28] Kelliher C, Anderson D. Doing more with less? Flexible working practices and the intensification of work. Human Relations. 2009;63(1):83–106. doi: 10.1177/0018726709349199

[29] Hobfoll SE. Conservation of resources. A new attempt at conceptualizing stress. Am Psychol. 1989;44(3):513–24. doi: 10.1037//0003-066x.44.3.513 2648906

[30] Bloom N, Han R, Liang J. Hybrid working from home improves retention without damaging performance. Nature. 2024;630(8018):920–5. doi: 10.1038/s41586-024-07500-2 38867040 PMC11208135

[31] Weerasinghe TD, Jayawardana AK. Flex-work and work-life balance: effects of role culture [Internet]. 2020.

[32] Mustajab D, Bauw A, Rasyid A, Irawan A, Akbar MA, Hamid MA. Working from home phenomenon as an effort to prevent COVID-19 attacks and its impacts on work productivity. TIJAB. 2020;4(1):13. doi: 10.20473/tijab.v4.i1.2020.13-21

[33] Magnusson C. Flexible time – but is the time owned? Family friendly and family unfriendly work arrangements, occupational gender composition and wages: a test of the mother-friendly job hypothesis in Sweden. Community, Work & Family. 2019;24(3):291–314. doi: 10.1080/13668803.2019.1697644

[34] Senik C, Clark AE, D’Ambrosio C, Lepinteur A, Schröder C. Teleworking and life satisfaction during COVID-19: the importance of family structure. J Popul Econ. 2024;37(1). doi: 10.1007/s00148-024-00979-z

[35] Ryan RM, Deci E. Self-determination theory. Encyclopedia of quality of life and well-being research. Springer Netherlands. 2014. 5755–60. doi: 10.1007/978-94-007-0753-5_2630

[36] Radcliffe J. Working from home: issues and strategies.

[37] Morgan RE. Teleworking: an assessment of the benefits and challenges. European Business Review. 2004;16(4):344–57. doi: 10.1108/09555340410699613

[38] Harpaz I. Advantages and disadvantages of telecommuting for the individual, organization and society. Work Study. 2002;51(2):74–80.

[39] Kunnen ES, Bosma HA. Fischer’s skill theory applied to identity development: A response to Kroger. Identity. 2003;3(3):247–70.

[40] Sarker AH, Kabir MF, Hossain KA, Jahan S, Hossain MZ, Hossain T, et al. Two-year epidemiology of post-COVID-19 conditions in Bangladesh: a cohort study of post-COVID-19 from 12,925 SARS-CoV-2 cases between July and December 2021–2023 in Bangladesh. Archives of Public Health. 2024;82(1):148. 39232821 10.1186/s13690-024-01358-6 PMC11373300

[41] Hayat MA, Chaudhry MA, Batool M, Ghulam H, Khan AR, Spulbar C, et al. Turning crisis into a sustainable opportunity regarding demand for training and new skills in labor market: an empirical analysis of COVID-19 pandemic and skills upgradation. Sustainability. 2022;14(24):16785. doi: 10.3390/su142416785

[42] Gaudet CH, Brown HQ, Lunsford DL. HRD curriculum meets global human capital challenge. Advances in Developing Human Resources. 2017;19(2):124–37. doi: 10.1177/1523422317695211

[43] Laleman F, Pereira V, Malik A. Understanding cultural singularities of ‘Indianness’ in an intercultural business setting. Culture and Organization. 2015;21(5):427–47. doi: 10.1080/14759551.2015.1060232

[44] Collewet M, Sauermann J. Working hours and productivity. Labour Economics. 2017;47:96–106. doi: 10.1016/j.labeco.2017.03.006

[45] Braun RL. The effect of time pressure on auditor attention to qualitative aspects of misstatements indicative of potential fraudulent financial reporting. Accounting, Organizations and Society. 2000;25(3):243–59. doi: 10.1016/s0361-3682(99)00044-6

[46] Tummers LG, Bakker AB. Leadership and job demands-resources theory: a systematic review. Frontiers in Psychology. 2021;12:722080. doi: 10.3389/fpsyg.2021.722080 34659034 PMC8514935

[47] Bick A, Blandin A, Mertens K. Work from Home before and after the COVID-19 Outbreak. American Economic Journal: Macroeconomics. 2023;15(4):1–39. doi: 10.1257/mac.20210061

[48] Hwang MI. The effectiveness of graphic and tabular presentation under time pressure and task complexity. Information Resources Management Journal. 1995;8(3):25–31. doi: 10.4018/irmj.1995070103

[49] Parkinson CN. Parkinson’s law. Economist. 1955.

[50] Yuan X, Wu Y, Sun L, Wang X. Research on efficient construction paths for intelligent coal mines in China from the configuration perspective. Applied Sciences. 2023;13(1):673. doi: 10.3390/app13010673

[51] Kniffin KM, Narayanan J, Anseel F, Antonakis J, Ashford SP, Bakker AB, et al. COVID-19 and the workplace: implications, issues, and insights for future research and action. American Psychologist. 2021;76(1):63. 32772537 10.1037/amp0000716

[52] Peng MW, Luo Y. Managerial ties and firm performance in a transition economy: the nature of a micro-macro link. Academy of Management Journal. 2000;43(3):486–501. doi: 10.2307/1556406

[53] Wut TM, Ng PM, Low MP. Engaging university students in online learning: a regional comparative study from the perspective of social presence theory. J Comput Educ. 2023;11(3):763–89. doi: 10.1007/s40692-023-00278-8

[54] Serenko A. The human capital management perspective on quiet quitting: recommendations for employees, managers, and national policymakers. JKM. 2023;28(1):27–43. doi: 10.1108/jkm-10-2022-0792

[55] Bowling NA. Organizational constraints as a source of work stress: a multi-facet perspective. Stress, Wellness, and Performance Optimization. Apple Academic Press. 2023. 1–20. doi: 10.1201/9781003400172-1

[56] Ellis BJ, Del Giudice M. Developmental adaptation to stress: an evolutionary perspective. Annu Rev Psychol. 2019;70:111–39. doi: 10.1146/annurev-psych-122216-011732 30125133

[57] Lee DY, Dawes PL. Guanxi, trust, and long-term orientation in Chinese business markets. Journal of International Marketing. 2005;13(2):28–56. doi: 10.1509/jimk.13.2.28.64860

[58] Wirawan H, Samad MA, Khairil M. Investigating the effect of abusive supervision on work engagement through the role of employee creativity: the moderating effect of interpersonal communication competence. Human Resource Development International. 2023;27(1):36–57. doi: 10.1080/13678868.2023.2193809

[59] Simbula S, Margheritti S, Avanzi L. Building work engagement in organizations: a longitudinal study combining social exchange and social identity theories. Behav Sci (Basel). 2023;13(2):83. doi: 10.3390/bs13020083 36829312 PMC9952149

[60] Richardson KM. Managing employee stress and wellness in the new millennium. J Occup Health Psychol. 2017;22(3):423–8. doi: 10.1037/ocp0000066 28150995

[61] Pinheiro M, Ivandic I, Razzouk D. The economic impact of mental disorders and mental health problems in the workplace. Mental health economics: The costs and benefits of psychiatric care. Cham: Springer International Publishing. 2017. 415–30.

[62] Jay K, Andersen LL. Can high social capital at the workplace buffer against stress and musculoskeletal pain?: Cross-sectional study. Medicine (Baltimore). 2018;97(12):e0124. doi: 10.1097/MD.0000000000010124 29561410 PMC5895355

[63] Chao M-C, Jou R-C, Liao C-C, Kuo C-W. Workplace stress, job satisfaction, job performance, and turnover intention of health care workers in rural Taiwan. Asia Pac J Public Health. 2015;27(2):NP1827–36. doi: 10.1177/1010539513506604 24174390

[64] Laguna M, Razmus W, Żaliński A. Dynamic relationships between personal resources and work engagement in entrepreneurs. J Occupat Organ Psyc. 2017;90(2):248–69. doi: 10.1111/joop.12170

[65] Marsh E, Perez Vallejos E, Spence A. Digital workplace technology intensity: qualitative insights on employee wellbeing impacts of digital workplace job demands. Front Organ Psychol. 2024;2. doi: 10.3389/forgp.2024.1392997

[66] Bachrach DG, Naskar ST, Patel PC, Levy P. Horizontal pay dispersion as social context for predicting employee performance outcomes associated with diagnostic‐ versus criterion‐referenced performance feedback. Applied Psychology. 2023;73(2):830–50. doi: 10.1111/apps.12458

[67] Tatom JA. The “Problem” of Procyclical Real Wages and Productivity. Journal of Political Economy. 1980;88(2):385–94. doi: 10.1086/260871

[68] Ilmakunnas P. Returns to workers and hours in finnish manufacturing. Empirical Economics. 1994;19(4):533–53. doi: 10.1007/bf01205814

[69] Shepard E, Clifton T. Are longer hours reducing productivity in manufacturing? International Journal of Manpower. 2000;21(7):540–53. doi: 10.1108/01437720010378999

[70] Dolton P, Howorth C, Abouaziza M. The Optimal Length of the Working Day. In 30th Annual Conference of the European Society for Population Economics. 2016.

[71] Lu SF, Lu LX. Do mandatory overtime laws improve quality? Staffing decisions and operational flexibility of nursing homes. Management Science. 2017;63(11):3566–85. doi: 10.1287/mnsc.2016.2523

[72] Tansky JW, Cohen DJ. The relationship between organizational support, employee development, and organizational commitment: an empirical study. Human Resource Dev Quarterly. 2001;12(3):285–300. doi: 10.1002/hrdq.15

[73] Runhaar P, Bouwmans M, Vermeulen M. Exploring teachers’ career self-management. Considering the roles of organizational career management, occupational self-efficacy, and learning goal orientation. Human Resource Development International. 2019;22(4):364–84. doi: 10.1080/13678868.2019.1607675

[74] Adaga EM, Egieya ZE, Ewuga SK, Abdul AA. Risk management in banking: a philosophical perspective. Malays J Hum Resour Manag. 2024;1(2):122–30. doi: 10.26480/mjhrm.02.2024.122.130

[75] Joshi A, Sekar S, Das S. Decoding employee experiences during pandemic through online employee reviews: insights to organizations. PR. 2023;53(1):288–313. doi: 10.1108/pr-07-2022-0478

[76] Pathomphatthaphan S, Das S, Jena LK. Agile SHRM practices and employee-organisational outcomes during new normal: evidence from India and Thailand. Journal of Organizational Effectiveness. 2024 May 13;11(2):347–74.

[77] Lakshmi Devi S, Das S, Gayathri Kumar AR, Keerthana AH, Sai Ram PC, Rakesh K. Influence of Artificial Intelligence-Based Skill Development Training on Employability. International Journal of Educational Reform. 2024. doi: 10.1177/10567879241238366

[78] Blanche MT, Blanche MJ, Durrheim K, Painter D. Research in practice: Applied methods for the social sciences. Juta and Company Ltd. 2006.

[79] Creswell JW, Miller DL. Determining validity in qualitative inquiry. Theory into practice. 2000;39(3):124–30.

[80] Koopmans L, Bernaards CM, Hildebrandt VH, van Buuren S, van der Beek AJ, de Vet HCW. Improving the Individual Work Performance Questionnaire using Rasch analysis. J Appl Meas. 2014;15(2):160–75. 24950534

[81] Podsakoff PM, MacKenzie SB, Lee J-Y, Podsakoff NP. Common method biases in behavioral research: a critical review of the literature and recommended remedies. J Appl Psychol. 2003;88(5):879–903. doi: 10.1037/0021-9010.88.5.879 14516251

[82] Hair JF, Black WC, Babin BJ, Anderson RE, Tatham RL. Multivariate data analysis.

[83] Byrne BM. Structural equation modeling with Mplus: Basic concepts, applications, and programming. Routledge. 2013.

[84] Kline RB. Promise and pitfalls of structural equation modeling in gifted research. Gifted Child Quarterly. 2023;84(1):5–15.

[85] Gerbing DW, Anderson JC. An Updated Paradigm for Scale Development Incorporating Unidimensionality and Its Assessment. Journal of Marketing Research. 1988;25(2):186. doi: 10.2307/3172650

[86] Diamantopoulos A, Siguaw JA. Introducing LISREL: A guide for the uninitiated.

[87] Fornell C, Larcker DF. Evaluating structural equation models with unobservable variables and measurement error. Journal of Marketing Research. 1981;18(1):39. doi: 10.2307/3151312

[88] Mulki JP, Jaramillo JF, Locander WB. Effect of ethical climate on turnover intention: linking attitudinal- and stress theory. J Bus Ethics. 2007;78(4):559–74. doi: 10.1007/s10551-007-9368-6

[89] Bagozzi RP, Yi Y. On the evaluation of structural equation models. JAMS. 1988;16(1):74–94. doi: 10.1007/bf02723327

[90] Bal M, Brookes A. How sustainable is human resource management really? An argument for radical sustainability. Sustainability. 2022;14(7):4219. doi: 10.3390/su14074219

[91] Sutton RI, Staw BM. What theory is not. Administrative Science Quarterly. 1995;40(3):371. doi: 10.2307/2393788

[92] Duxbury L, Higgins C, Lee C. Work-family conflict: a comparison by gender, family type, and perceived control. Journal of Family Issues. 1994;15(3):449–66.

[93] Makridis C. Measuring the ins and outs of remote work: New evidence from the Gallup workplace panel. 2025. https://ssrn.com/abstract=5938995

[94] Marzocchi I, Nielsen K, Di Tecco C, Vignoli M, Ghelli M, Ronchetti M, et al. Job demands and resources and their association with employee well-being in the European healthcare sector: a systematic review and meta-analysis of prospective research. Work & Stress. 2024;38(3):293–320. doi: 10.1080/02678373.2024.2308812

[95] Reay T, Whetten DA. What constitutes a theoretical contribution in family business? Family Business Review. 2011;24(2):105–10. doi: 10.1177/0894486511406427

[96] Homer ST, Lim WM. Theory development in a globalized world: Bridging “Doing as the Romans Do” with “Understanding Why the Romans Do It”. Glob Bus Org Exc. 2023;43(3):127–38. doi: 10.1002/joe.22234

[97] Pickert R. Hybrid work reduced attrition rate by a third, study shows. 2022. Accessed 2022 October 6. https://www.bloomberg.com/news/articles/2022-07-25/hybrid-work-reduced-attrition-rate-by-a-third-new-study-shows?sref=QnKyEnuc&leadSource=uverify%20wall

[98] Drapkin A. Apple finally starts hybrid work pilot, a year after announcing it. Tech co. 2022.

[99] Fund SD. Sustainable development goals. 2015. https://www.un.org/sustainabledevelopment/inequality

[100] Moglia M, Hopkins J, Bardoel A. Telework, hybrid work and the United Nation’s sustainable development goals: towards policy coherence. Sustainability. 2021;13(16):9222. doi: 10.3390/su13169222

